# Synthesis, Stability Studies, and Antifungal Evaluation of Substituted α- and β-2,3-Dihydrofuranaphthoquinones against *Sporothrix brasiliensis* and *Sporothrix schenckii*

**DOI:** 10.3390/molecules24050930

**Published:** 2019-03-07

**Authors:** Patricia Garcia Ferreira, Luana Pereira Borba-Santos, Leticia Lorena Noronha, Caroline Deckman Nicoletti, Marcella de Sá Haddad Queiroz, Fernando de Carvalho da Silva, Sônia Rozental, Débora Omena Futuro, Vitor Francisco Ferreira

**Affiliations:** 1 Departamento de Tecnologia Farmacêutica, Faculdade de Farmácia, Universidade Federal Fluminense, Niterói-RJ 24241-000, Brazil; patricia.pharma@yahoo.com.br (P.G.F.); leticianoronha95@gmail.com (L.L.N.); caroline_nicoletti@yahoo.com.br (C.D.N.); marcellahaddad@id.uff.br (M.d.S.H.Q.); dfuturo@id.uff.br (D.O.F.); 2 Laboratório de Biologia Celular de Fungos, Instituto de Biofísica Carlos Filho, Universidade Federal do Rio de Janeiro, Rio de Janeiro 21941-902, RJ-Brazil; luana.p.borba@gmail.com (L.P.B.-S.); rozental@biof.ufrj.br (S.R.); 3 Departamento de Química Orgânica, Instituto de Química, Universidade Federal Fluminense, Niterói-RJ 24210-141, Brazil; gqofernando@vm.uff.br

**Keywords:** furanophthoquinones, antifungal, *Sporothrix brasiliensis*, *Sporothrix schenckii*, stability study

## Abstract

Sporotrichosis is a neglected fungal infection caused by *Sporothrix* spp., which have a worldwide distribution. The standard antifungal itraconazole has been recommended as a first-line therapy. However, failure cases in human and feline treatment have been reported in recent years. This study aimed to synthesize several α- and β-2,3-dihydrofuranaphthoquinones and evaluate them against *Sporothrix schenckii* and *Sporothrix brasiliensis*—the main etiological agents of sporotrichosis in Brazil. The stability of these compounds was also investigated under different storage conditions for 3 months. The samples were removed at 0, 60, and 90 days and assessed by ^1^H-NMR, and their in vitro antifungal susceptibility was tested. Furthermore, we evaluated the superficial changes caused by the most effective and stable compounds using scanning electron microscopy and determined their effects when combined with itraconazole. Nine dihydrofuranaphthoquinones showed good antifungal activity and stability, with MIC values of 2–32 µM. Compounds **6** and **10** were the most active dihydrofuranaphthoquinones in vitro for both species; in fungi, these compounds induced yeast–hyphae conversion and alteration in the hyphae and conidia structures. Compound **10** also exhibited a synergistic activity with itraconazole against *S. schenckii*, with a ΣFIC index value of 0.3. Our results indicate that Compounds **6** and **10** are potential candidates for the development of new antifungal agents for the treatment of sporotrichosis.

## 1. Introduction

Fungal infections are opportunistic and neglected diseases that are difficult to treat and are therefore a serious global public health problem for humans, plants, and animals. It is estimated that there are approximately 1.5 million species of fungi, of which 8000 are known to cause diseases in plants and more than 300 affect humans [1]. These infections affect more than one billion people worldwide and can cause more than 1.5 million deaths annually [2,3]. *Candida*, *Aspergillus*, *Pneumocystis*, and *Cryptococcus* are the most common fungi species that cause serious diseases in humans and lead to morbidity and mortality, especially among severely ill or immunocompromised patients or those with complicated medical conditions [4,5]. Resistance of fungi to antifungal drugs poses another threat to public health, food biosafety, and biodiversity.

The development of new drugs, diagnostic kits, and antifungal therapies has received little attention from development agencies and pharmaceutical industries, particularly when compared with bacterial and viral infections.

Among neglected mycoses, sporotrichosis is a subacute or chronic disease that affects humans and several animals, especially felines [6]. Sporotrichosis is caused by several thermodimorphic fungi species of the *Sporothrix* genus, such as *Sporothrix brasiliensis, Sporothrix schenckii, Sporothrix globosa, Sporothrix mexicana*, and *Sporothrix luriei* [7].

The *S. schenckii* is the main etiological agent of sporotrichosis in the world [8], but in Brazil, *S. brasiliensis* prevails as the main etiological agent of human and animal sporotrichosis [9,10,11]. Currently, *S. brasiliensis* has become the most frequent species associated with feline and human cases of sporotrichosis in the country [12]. The infection is usually acquired by the traumatic inoculation of this dimorphic fungus by suppurating subcutaneous nodules that slowly progress along the lymphatic channels [13].

**Figure 1:**
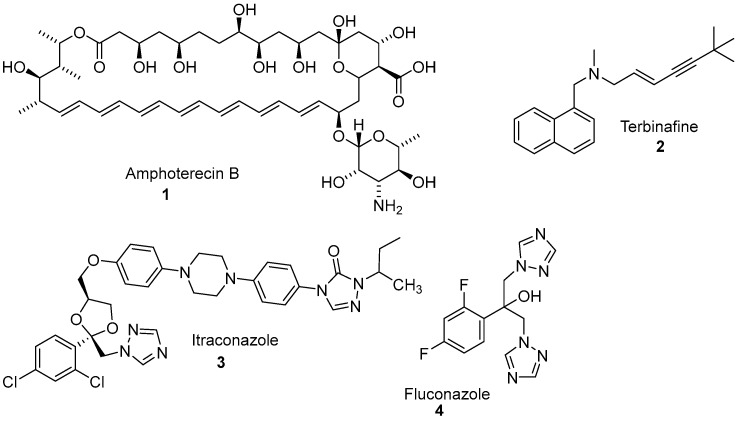
Some drugs currently used in the treatment of sporotrichosis.

In the past, cutaneous and lymphocutaneous sporotrichosis infections have been treated with saturated solutions of potassium iodide, which have low effectiveness and present adverse effects [14]. Currently, some more effective antifungal drugs, such as amphotericin B (**1**), terbinafine (**2**), itraconazole (**3**), and fluconazole (**4**) [15] are in the therapeutic arsenal (Figure 1) [16,17,18,19]. In this regard, itraconazole (**3**) is the most common and effective drug used for sporotrichosis infection in clinical treatments [8]. However, it is important to note that several failure cases have already been reported in both human and feline treatment [20,21].

The discovery of novel, effective, and stable antifungal agents is a long-term process with high cost. Therefore, the search for new antifungal agents is extremely necessary and urgent. Regarding the *Sporothrix* infection, few synthetic compounds [22,23,24], natural products [25], and crude extracts [26,27] have been investigated.

Natural products have been an important source of bioactive substances and have been an inspiration for the synthesis of new drug prototypes. Among these products, naphthoquinones are widely distributed in plants, lichens, bacteria, and higher animals, and they perform functions in vital biochemical processes. Over the last few years, this class of compounds has gained remarkable attention due to their diversified biological activities, including their bactericidal [28], leishmanicidal [29], antimalarial [30], tripanocidal [31], antitubercular [32], anticancer, and antifungal [33] effects. Their mechanism of action is related to redox cycles, arylation of the thiol groups of proteins, intercalation, the induction of breaks in the DNA chain, the generation of free radicals and other reactive oxygen species (ROS), and bioreductive alkylation via the formation of quinone methide. A number of studies have demonstrated that substituted pyranaphthoquinones [34] show a particular activity against *Candida* species and dermatophytes fungi [35,36]. However, there are few studies about natural and synthetic naphthoquinones against the *Sporothrix* genus [24,37,38] as well as their sensitivity to temperature [39]—an important information that would allow their manufacture, formulation, storage, and administration.

Tandon and coworkers reported the synthesis of some hetero-1,4-naphthoquinone derivatives and evaluated their antifungal and antibacterial activities, including those against *S. schenckii*. The results indicated that several derivatives had potent antifungal activity compared to clinically prevalent antifungal drugs against *S. schenckii*, reaffirming the importance of quinones as a drug candidate [24,40,41,42,43,44,45].

Considering our preliminary studies involving α- and β-pyran and α- and β-furanaphthoquinones against various biological targets as well as our research program on the synthesis of biologically active naphthoquinones [35,46], we became interested in the synthesis of substituted α- and β-2,3-dihydrofuranaphthoquinones and their in vitro antifungal activities against *S. brasiliensis* and *S. schenckii*. For this purpose, we synthesized 17 naphthoquinones and evaluated their chemical stability. We determined the inhibitory activities of these compounds and compared them with itraconazole (**3**). The most effective and stable naphthoquinones were selected, and we evaluated their effect on fungal morphology, their interaction with itraconazole (**3**), and their hemolytic activity.

It is well established that, in addition to the search of new molecules, it is important to investigate the safety and effectiveness of these compounds at different temperatures, which can compromise a possible candidate drug. Therefore, the evaluation of the stability of drug products is an essential stage in the formulation development process of the pharmaceutical industry and is an important tool for obtaining information for clinical trials for the evaluation of new drug registration. The purpose of the stability test is to provide evidence of how the quality of a product changes with time under the influence of various environmental factors such as temperature, humidity, and light [47].

## 2. Experimental Methods

### 2.1. Materials

Itraconazole (Sigma Chemical, Co., St. Louis, MO, USA) and α- and β-2,3-dihydrofuranaphthoquinones were diluted in dimethyl sulfoxide (DMSO) to obtain stock solutions of 1 mM and 50 mM, respectively. Compounds were kept at −20 °C until testing, when they were diluted in RPMI 1640 medium supplemented with 2% glucose, and buffered with 0.165 M 3-(*N*-morpholino)propanesulfonic acid at pH 7.2.

### 2.2. Synthesis of α- and β-2,3-Dihydrofuranaphthoquinones ***5**–**21***

**Scheme 1:**
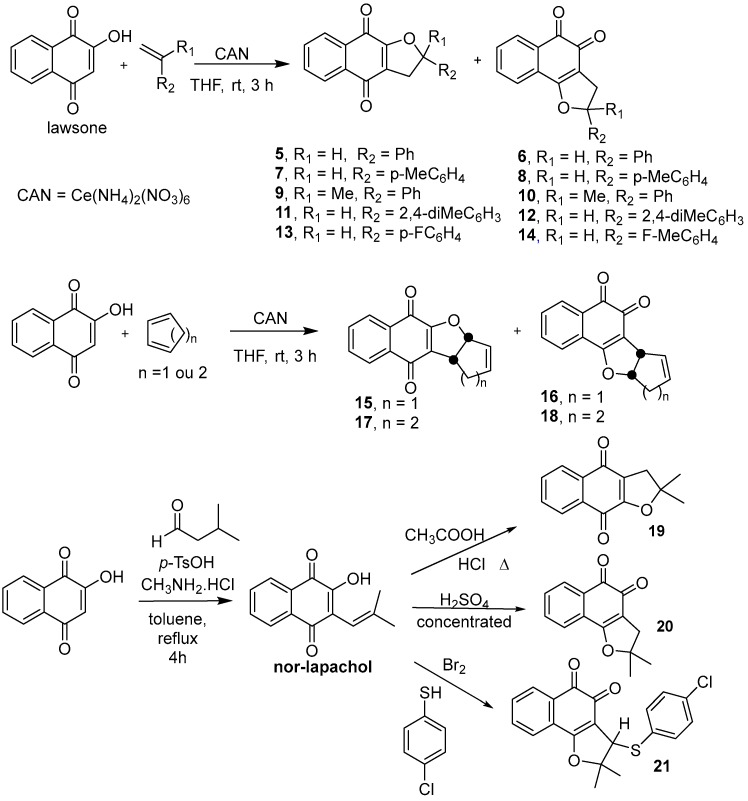
Synthetic routes for preparing α- and β-2,3-dihydrofuranaphthoquinones **5**–**21**.

All the dihydrofuranaphthoquinones used for antifungal screening against *S. brasiliensis* and *S. schenckii* were prepared from lawsone by different methods (Scheme 1) with a yield of 23–76%. The α- and β-2,3-dihydrofuranaphthoquinones **5**–**18** were prepared as previously described in the literature [35,48] and were confirmed by ^1^H-NMR spectra recorded on a Varian VNMR (500 MHz) spectrometer using chloroform-d as a solvent and tetramethylsilane as an internal standard. The α- and β-2,3-dihydrofuranaphthoquinones **19**–**21** were prepared from nor-lapachol, as previously described [49,50].

### 2.3. Yeast Sources and Manipulation

*S. brasiliensis* CBS 133,006 and *S. schenckii* ATCC 3228 yeast strains were used in this study and were stored in a saline solution with 10% glycerol and 10% glucose at −20 °C until use. The conversion of the isolate to the yeast (pathogenic form) was performed from the filamentous form (saprophytic form). Mycelium forms were cultivated on Sabouraud broth at 36 °C for 7 days. The conversion to the yeast phase was achieved by adding 10^5^ conidia/mL to brain heart infusion (Difco) broth supplemented with 2% glucose, pH 7.8 at 36 °C with orbital agitation (150 rpm) for 7 days.

### 2.4. Stability Study

A stability study of α- and β-2,3-dihydrofuranaphthoquinones was conducted for 3 months under different storage conditions. The samples were placed into 2 mL Eppendorf and then stored at ambient room temperature (25 °C) and under refrigeration (−20 °C). Samples were taken at 0, 60, and 90 days and assessed by ^1^H-NMR and antifungal activity.

### 2.5. Susceptibility Assays

#### 2.5.1. Antifungal Assay of Compounds **5**–**21**

α- and β-2,3-Dihydrofuranaphthoquinones and itraconazole (standard) were diluted in RPMI 1640 medium (supplemented with 2% glucose and buffered at pH 7.2, and 0.165 M 3-(*N*-morpholino)propanesulfonic acid (MOPS)) and added into flat-bottom 96-well microplates to obtain a final concentration of 32 µM. *Sporothrix* yeasts were added into a microtiter plate at a final yeast concentration ranging from 0.5–1 × 10^5^ ufc/mL; treatments were performed for 48 h at 35 °C in a dark 5% CO_2_ chamber. Fungal growth was evaluated by visual inspection in an inverted light microscope and quantified by spectrophotometric readings at 492 nm in a microtiter plate reader (Emax ELISA plate reader Molecular Devices, Sunnyvale, CA, USA).

#### 2.5.2. Minimum Inhibitory Concentration Determination

The minimum inhibitory concentrations (MIC) of α- and β-2,3-dihydrofuranaphthoquinones **5**–**21** were determined by the broth microdilution test as described in the Clinical and Laboratory Standards Institute (CLSI) protocols M27-A3 [51], with minor adaptations for the use of *Sporothrix* spp. yeasts. The compounds were diluted in RPMI 1640 medium buffered at pH 7.2 with 0.165 M MOPS with 2% glucose into 96-well plates to obtain final concentrations of 0.06–32 µM for naphthoquinones and 0.002–1 µM for itraconazole (**3**). Then, 100 µL of the fungal suspension was dispensed into each well for a final yeast cell density of 0.5–1.5 × 10^5^ colony forming units (CFU)/mL. The samples were incubated for 48 h in the dark in a humid chamber with 5% CO_2_. The fungal growth inhibition was observed visually with an inverted optical microscope. Subsequently, the samples were homogenized, and the OD was measured using a microplate reader (Emax ELISA plate reader Molecular Devices, Sunnyvale, CA, USA) at 492 nm. MIC corresponds to the lowest drug concentration that inhibits 50% of fungal growth relative to untreated controls.

#### 2.5.3. Combination Assay

The effects of interactions between the most effective α- and β-2,3-dihydrofuranaphthoquinones **6** and **10** and itraconazole (**3**) were evaluated using the checkerboard microdilution method [52]. Concentrations tested ranged from 0.25 to 16 µM or from 0.001 to 1 µM for the dihydrofuranaphthoquinones and itraconazole, respectively. Yeasts (0.5–1 × 10^5^ CFU/mL) were treated for 48 h at 35 °C in a 5% CO_2_ chamber. The MIC values for individual compounds and for their combinations were determined, and a better combination was defined as the lowest value of the fractional inhibitory concentration index (ΣFIC index), which is calculated using the equation: ΣFIC index = (MIC_naphthoquinone_ in combination/MIC_naphthoquinone_ alone) + (MIC_itraconazole_ in combination/MIC_itraconazole_ alone) [53]. Interactions were considered to be synergistic if FICI ≤ 0.5 [52].

### 2.6. Scanning Electron Microscopy

Yeasts were incubated with a sub-inhibitory concentration (1/2 MIC) of the most effective naphthoquinones at 36 °C with orbital agitation for 48 h. Untreated and treated cells were washed three times in phosphate buffered saline (PBS) and fixed in a solution of 2.5% glutaraldehyde and 4% formaldehyde in a 0.1 M cacodylate buffer for 24 h at 4 °C. The cells were plated on poly-l-lysine-covered glass coverslips and postfixed in 1% osmium tetroxide in a 0.1 M cacodylate buffer containing 1.25% potassium ferrocyanide and 5 mM CaCl_2_ for 30 min. Samples were washed, dehydrated in increasing ethanol concentrations (30% to ultradry), critical-point-dried in CO_2_, and coated with gold. The images were obtained using a Quanta FEC 250 scanning electron microscope (Thermo Scientific, Hillsboro, OR, USA).

### 2.7. Hemolytic Assay

Human red blood cells (RBCs) were collected in accordance with an approved protocol from the Human Research Ethics Committee of Hospital Universitário Clementino Fraga Filho, UFRJ (protocol number # 50765015.5.0000.5257). Solutions of the most effective naphthoquinones were prepared in DMSO to obtain stock solutions of 100 mM and were diluted in a saline solution to different concentrations. Naphthoquinones were added to microplate wells to obtain final concentrations ranging from 0.5 to 1000 μM. RBC suspensions were then prepared in a saline solution (0.9% NaCl in ultra-pure water, pH 7.2) and added to microplate wells at a final density of 0.5%. RBCs were incubated in the presence of naphthoquinones for 1 h at 37 °C before centrifuging at 3000 rpm for 15 min and transferring the supernatant to a new microplate. The absorbance of hemoglobin released from RBCs was measured at 562 nm using a microtiter plate reader (EMax Plus, Molecular Devices), and the concentration that lysed 50% of RBCs (HA_50_) was determined [35].

### 2.8. Thermal Analyses

Differential scanning calorimetry (DSC) analyses were performed using a SHIMADZU differential scanning calorimeter DSC-60A (SHIMADZU, Columbia, SC, USA.) thermal analyzer in a nitrogen dynamic atmosphere with a flow rate of 50 mL. min^−1^ at a heating rate of 10 °C min^−1^ over the range of 25–300 °C; for DSC analyses, 3.0 mg of sample was evaluated in a closed aluminum cell. Thermogravimetric (TG) analyses were performed using a NETZSCH STA 409 PC/PG (Netzsch-Gerätebau GmbH, Selb, Germany) in a nitrogen dynamic atmosphere with a flow rate of 60 mL min^−1^ and a heating rate of 10 °C min^−1^ over the range of 30–300 °C; for TG analyses, 6 mg of sample was evaluated in an aluminum cell. An empty aluminum cell was used as a reference for both analyses.

## 3. Results and Discussion

**Figure 2:**
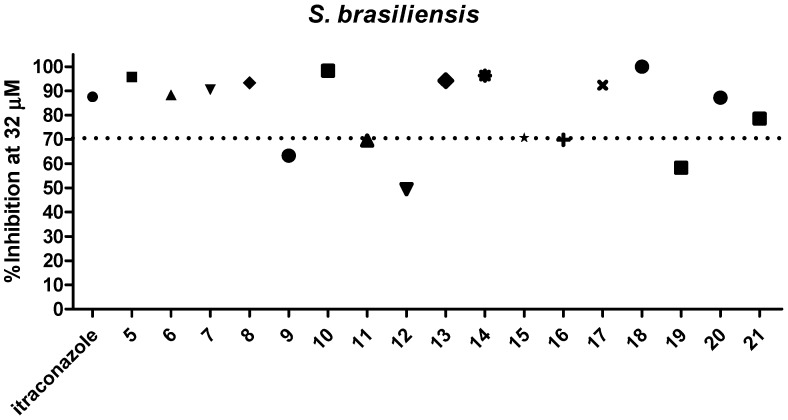
Evaluation of **5**–**21** against *S. brasiliensis* growth at 32 µM.

**Figure 3:**
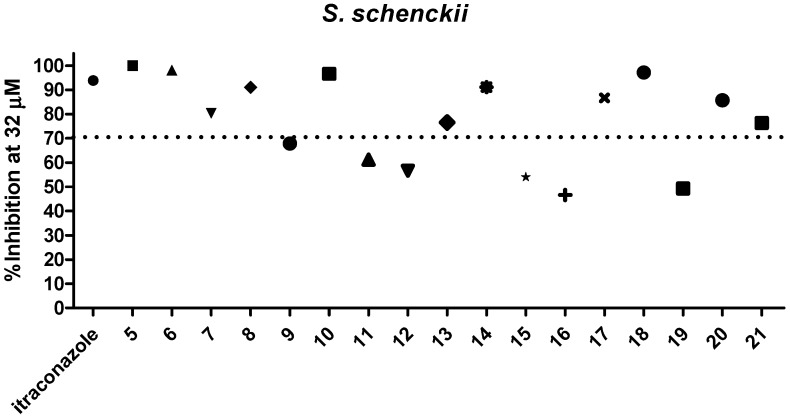
Evaluation of **5**–**21** against *S. schenckii* growth at 32 µM.

Dihydrofuranaphthoquinones **5**–**21** were prepared and evaluated in a preliminary inhibitory screening against *S. brasiliensis* and *S. schenckii* growth at 32 µM, as shown in Figure 2 and Figure 3, respectively. All compounds evaluated were able to inhibit the growth of both fungi. From these preliminary tests, it was observed that only one compound (**12**) had activity below 50%, two compounds (**9** and **19**) had activity between 60 and 70%, three compounds (**11**, **15**, and **16**) had activity of approximately 70%, and eleven compounds (**6**, **7**, **8**, **10**, **13**, **14**, **15**, **17**, **18**, **20**, and **21**) were able to inhibit the fungi growth by more than 70% and were similar in activity to itraconazole (*p* > 0.05) against both species. Among the most active compounds, three were α-dihydrofuranaphthoquinones (**7**, **13**, and **17**), but eight were the β-dihydrofuranaphthoquinone isomers (**6**, **8**, **10**, **14**, **15**, **18**, **20**, and **21**), indicating that these isomers were the most active series as shown in Figure 1, Figure 2, and Table 1, respectively. The concentration of 32 μM was selected for this preliminary test due to the precipitation of naphthoquinones in a more concentrated medium.

After preliminary evaluation as reported in Figure 2 and Figure 3, the active derivatives were submitted to a broth microdilution assay to determine the lowest concentration capable of inhibiting fungi growth by 50% (MIC—minimum inhibitory concentration). Additionally, a stability study of the dihydrofuranaphthoquinones vs. antifungal activity was performed for different time periods and at different storage temperatures. Samples were also analyzed by ^1^H-NMR spectroscopy (Table 2).

The MIC values obtained for selected compounds at T0 indicate that β-dihydrofuranaphthoquinone isomers have higher antifungal activities against both *Sporothrix* species when compared to their α-dihydrofuranaphthoquinone isomers. This is most evident when the MIC values of the compounds **6**, **8**, and **18** are compared with those of their respective isomers α-dihydrofuranaphthoquinones **5**, **7**, and **17**.

The samples were submitted to a stability study and were analyzed by ^1^H-NMR at T0, T60, and T90; their spectra showed no changes. The biological activity of samples stored at room temperature and under refrigeration showed some variations. One of the most distinct features observed in Table 2 is that Compounds **6** and **10** exhibited the lowest MIC values against *S. brasiliensis* and *S. schenckii* after 90 days under both storage conditions. The ^1^H-NMR spectra of compounds **6** and **10** were used in the study of their stabilities and are available in the supplementary materials. However, their antifungal activity was not higher than itraconazole, which displayed MIC values of 0.25 and 0.125 µM for *S. brasiliensis* and *S. schenckii*, respectively.

**Figure 4:**
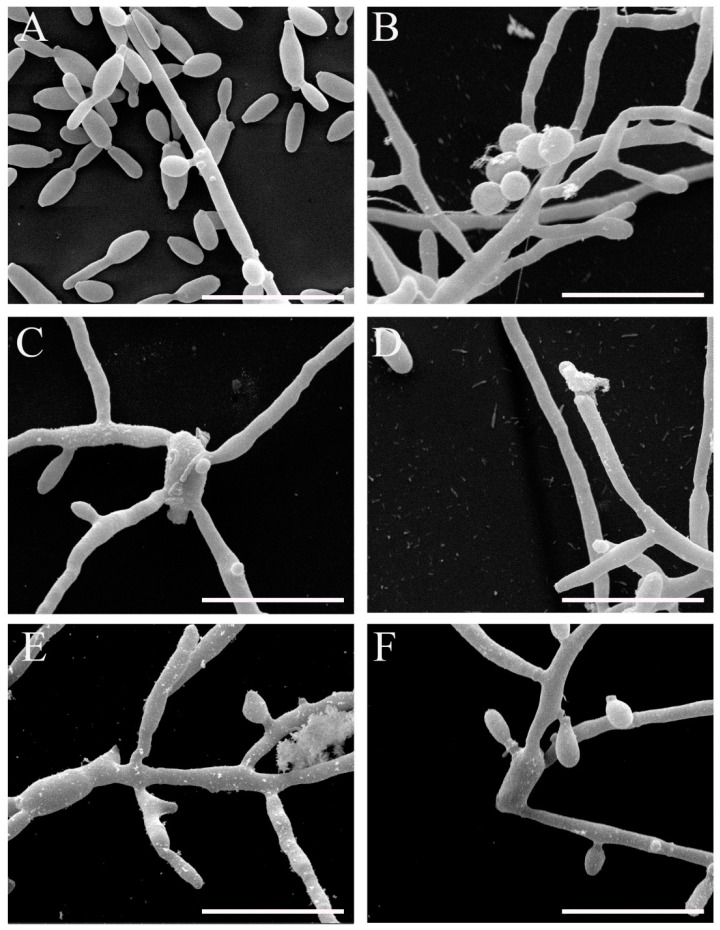
Scanning electron microscopy images of *S. brasiliensis* (**A**,**C**,**E**) and *S. schenckii* (**B**,**D**,**F**) after exposure to sub-inhibitory concentrations of Compound **10** (**C** and **D**) and Compound **6** (**E** and **F**) compared to untreated cells (**A** and **B**) (bars = 10 µm).

We also analyzed the superficial changes in the *Sporothrix* cells induced by β-2,3-dihydrofuranaphthoquinones **6** and **10**. The fungi were treated with sub-inhibitory concentrations of these compounds for 48 h, and cells were visualized by scanning electron microscopy. Untreated *S. brasiliensis* exhibited the most cells in yeast form, with oval or elongated cells showing multiple budding and some hyphae (Figure 4A); the untreated *S. schenckii* showed mainly filamentous morphology, with thin hyphae and conidia adhered (Figure 4B). *S. brasiliensis* treated with 1 µM of Compound **10** showed cells mainly in a filamentous form with an altered morphology (Figure 4C). *S. schenckii* treated with 2 µM of Compound **10** exhibited altered hyphae with a ruptured cell wall (Figure 4D). Treatment of both species with 2 µM of Compound **6** led to the alteration in hyphae morphology and conidia structure, mainly *S. brasiliensis* cells (Figure 4E,F). These results indicate that, even at sub-inhibitory concentrations, the compounds were able to induce damage in *Sporothrix* spp.

In view of the high effectiveness and stability of β-2,3-dihydrofuranaphthoquinones **6** and **10**, we decided to test whether these compounds could improve itraconazole (**3**) activity. Interactions with the ΣFIC index <0.5 were categorized as synergistic, and those with ΣFIC index values from >0.5 to 4 were categorized as having no interaction [53]. As shown in Table 3, Compound **10** exhibited a synergistic activity with itraconazole against *S. schenckii* with a ΣFIC index value of 0.3 *i*. Importantly, it should be noted that the MIC of itraconazole was reduced when *S. schenckii* was coincubated with Compound **6**.

Finally, the hemolytic activities of Compounds **6** and **10** were studied, and no hemolytic effect toward RBCs was observed for either compound at concentrations up to 1000 µM. Thus, these compounds were more selective for the fungi because much smaller concentrations affected the fungi when compared to the RBCs. These results also confirm that Compounds **6** and **10** do not affect the plasma membrane of eukaryotic cells; if this happened, we would see hemolysis at low concentrations.

Thermogravimetry (TG) and differential scanning calorimetry (DSC) are techniques with potential for application in pharmaceutical research to determine the physicochemical properties of compounds such as polymorphism, purity, and thermal stability. In addition, they are essential for preformulation studies because they provide important information about the physical characteristics or chemical interactions between drugs and excipients [54,55]. Therefore, it is important to study thermal degradation and, consequently, to evaluate the quality control of Compounds 6 and 10 using techniques such as TG and DSC. The difference between the two techniques is that TG measures mass variation, whereas DSC allows for the measurement of changes in enthalpy. Both analyses are performed using a heating program [56].

**Figure 5:**
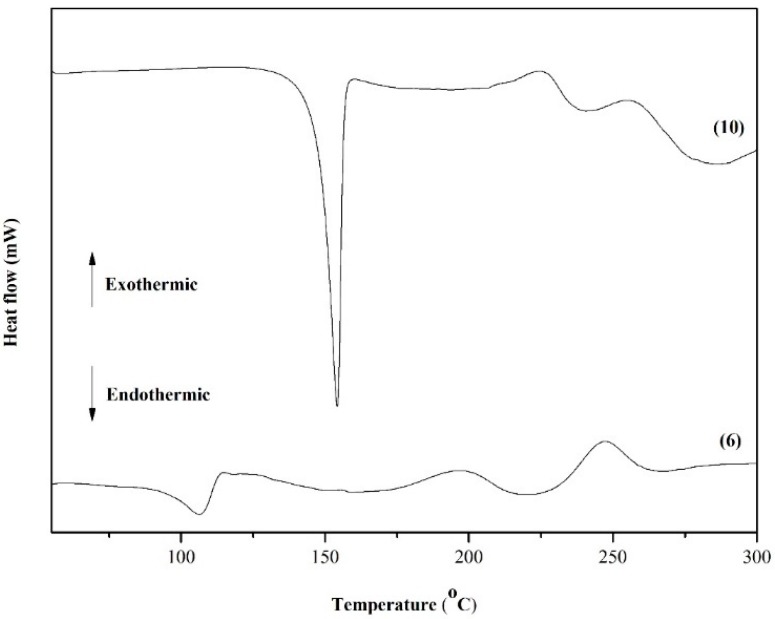
DSC curves of dihydrofuranaphthoquinones **6** and **10**.

**Figure 6:**
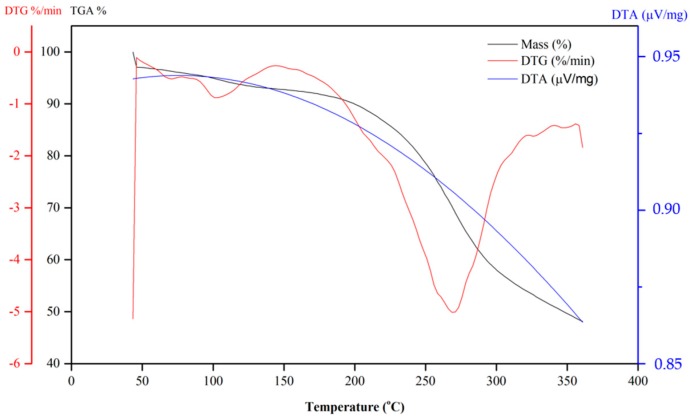
TG curves of dihydrofuranaphthoquinones **6**.

Figure 5 shows the DSC curves obtained for quinones **6** and **10**. The TGA and DTG curves of Compound **6** show that the naphthoquinone is thermally stable up to 200 °C. According to the TG curve of Compound **6** (Figure 6), in the first step, the quinone lost approximately 6.2% of mass between 100 and 120 °C and continued to lose mass. This initial mass loss is associated with an endothermic peak at 110 °C in the DSC curve (Figure 5), corresponding to the melting of the compound. The fusion is characterized as a physical phenomenon, which can be detected through DSC or DTA curves, and presents itself as an endothermic event [57]. The additional mass loss of approximately 20% associated with a set of successive exothermic and endothermic peaks at 200 and 225 °C, respectively, in the DSC curves may be due to the decomposition of the compound. At higher temperatures (above 225 °C), the peaks may be attributed to the pyrolysis of the compound.

**Figure 7:**
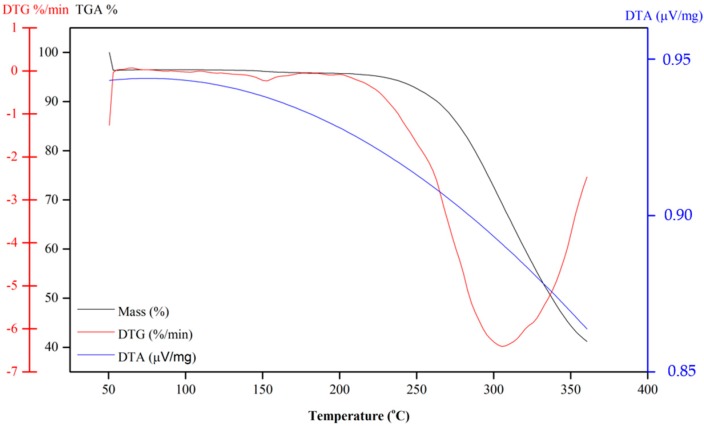
TG curves of dihydrofuranaphthoquinones **10**.

Compound **10** also shows continual mass loss between 200 and 350 °C (Figure 7). This mass loss is associated with an endothermic peak at 150 °C in the DSC curve (Figure 5), which is also related to the fusion of the compound. In summary, according to TG and DSC curves, Compound **6** is thermally stable only until approximately 150 °C. Above this temperature, the naphthoquinone is decomposed. An exothermic peak at 200 °C may be due to the decomposition of the compound, which is followed by another exothermic peak at 250 °C that is likely attributed to the pyrolysis of the compound.

## 4. Conclusions

In conclusion, we synthesized a series of substituted α- and β-2,3-dihydrofuranaptoquinones, which were confirmed by 1H-NMR data and are consistent with those reported in the literature. The antifungal profile indicated that Compounds **6** and **10** presented potent antifungal activity against the main etiological agents of sporotrichosis in Brazil—*S. brasiliensis* and *S. schenckii*. Studies indicate the existence of several strains of *S. brasiliensis* and *S. schenckii* that are resistant to itraconazole, and this fact led us to evaluate the effect of Compounds **6** and **10** in combination with itraconazole. Compound **10** showed a synergistic action against itraconazole against *S. schenckii* resulting in a reduction of MIC, showing that this is a promising strategy for the treatment of Sporotrichosis with the use of itraconazole and naphthoquinone combinations. In addition, it was possible to highlight the main thermal events of these compounds, which is very important for the quality control of a candidate drug or drug combination. This work opens perspectives for future studies in the attempt to discover new antifungal agents against *S. brasiliensis* and *S. schenckii* based on naphthoquinone framework.

## Figures and Tables

**Table 1:** % Inhibition at 32 µM of naphthoquinones **5**–**20**.

Compounds	%Inhibition at 32 µM ^a^	
	*S. brasiliensis*	*S. schenckii*
**Itraconazole**	87.5 ± 12.5	93.8 ± 2.6
**5**	95.7 ± 3.1	100.0 ± 0.0
**6**	88.3 ± 12.8	98.2 ± 2.4
**7**	90.5 ± 7.5	80.3 ± 22.4
**8**	93.3 ± 11.2	91.0 ± 12.5
**9**	63.3 ± 16.0 *	67.8 ± 19.5 *
**10**	98.3 ± 1.9	96.7 ± 4.7
**11**	69.5 ± 13.4	61.2 ± 18.1 **
**12**	49.5 ± 21.8 ***	56.7 ± 6.8 ***
**13**	94,2 ± 11.4	76.5 ± 19.9
**14**	96.3 ± 5.7	91.2 ± 9.8
**15**	70.7 ± 17.0	54.0 ± 7.5 ***
**16**	69.8 ± 14.5	46.7 ± 8.2 ***
**17**	92.3 ± 6.1	86.7 ± 10.6
**18**	100.0 ± 0.0	97.2 ± 2.2
**19**	58.3 ± 18.5 **	49.3 ± 24.6 ***
**20**	87.2 ± 19.2	85.7 ± 15.5
**21**	78.5 ± 13.7	76.3 ± 24.0

^a^ Mean ± Standard Deviation of two independent experiments, made in triplicate. * *p* < 0.05, ** *p* < 0.001, *** *p* < 0.0001 vs. treatment with itraconazole (by one-way ANOVA and Dunnett’s test).

**Table 2:** Antifungal activity of dihydrofuranaphthoquinones over time and at different storage temperatures against *S. brasiliensis* and *S. schenckii* fungi.

DihydrofuraNa-Phthoquinone	Time and Condition	MIC (µM)	
	Time (day)	*S. brasiliensis*	*S. schenckii*
**5**	0	16	32
	60, refrigerator	8	16
	60, room temperature	16	16
	90, refrigerator	16	16
	90, room temperature	8	4
**6**	0	4	4
	60, refrigerator	4	4
	60, room temperature	4	4
	90, refrigerator	4	4
	90, room temperature	4	4
**7**	0	32	32
	60, refrigerator	8	16
	60, room temperature	8	16
	90, refrigerator	16	16
	90, room temperature	16	16
**8**	0	4	8
	60, refrigerator	8	8
	60, room temperature	16	32
	90, refrigerator	16	16
	90, room temperature	16	16
**10**	0	2	4
	60, refrigerator	4	4
	60, room temperature	4	4
	90, refrigerator	4	4
	90, room temperature	16	8
**17**	0	16	32
	60, refrigerator	16	16
	60, room temperature	16	16
	90, refrigerator	>32	>32
	90, room temperature	>32	>32
**18**	0	4	8
	60, refrigerator	4	8
	60, room temperature	4	8
	90, refrigerator	8	8
	90, room temperature	8	8
**20**	0	16	16
	60, refrigerator	16	16
	60, room temperature	16	16
	90, refrigerator	>32	32
	90, room temperature	>32	32
**21**	0	32	16
	60, refrigerator	4	8
	60, room temperature	4	8
	90, refrigerator	4	8
	90, room temperature	4	8
Itraconazole		0.25	0.125

MIC: minimum inhibitory concentration. The results represent two independent experiments in duplicate. T0 = 0 days; T60 = 60 days; T90 = 90 days.

**Table 3:** Associations of itraconazole and Compounds **6** and **10** against *S. schenckii* and *S. brasiliensis* yeast cells. MIC values are in µM.

	**MIC Drugs Tested Alone**		**MIC ^b^ Drugs in Combination**	**ΣFIC Index** **^a^**
	**Itraconazole**	**6**	**Itraconazole/6**	
*S. brasiliensis*	0.25	4	0.25/0.5	1.13
*S. schenckii*	0.125	4	0.06/0.25	0.54
	**Itraconazole**	**10**	**Itraconazole/10**	
*S. brasiliensis*	0.25	2	0.25/0.25	1.13
*S. schenckii*	0.125	4	0.03/0.25	0.30

^a^ Fractional inhibitory concentration index; ΣFIC index = (MICa in combination/MICa tested alone) + (MICb in combination/MICb tested alone), where a and b are itraconazole and naphthoquinone (**10** or **6**), respectively. ^b^ Combinations with the lowest ΣFIC index. MIC was the lowest concentration able to inhibit 50% of fungal growth (mean of two independent experiments).

## Supplementary Materials

Supplementary Materials are available online.
