# Supplementary material for: Synthesis, Stability Studies, and Antifungal Evaluation of Substituted α- and β-2,3-Dihydrofuranaphthoquinones against Sporothrix brasiliensis and Sporothrix schenckii
Source: Molecules. 2019 Mar 7;24(5):930. doi: 10.3390/molecules24050930 (PMC6429059; doi:10.3390/molecules24050930)
Supplement: Supplementary file 1 [file molecules-24-00930-s001.pdf]

Synthesis, stability studies and antifungal evaluation of substituted α- and β-2,3-dihydrofuranaphthoquinones against *Sporothrix brasiliensis* and *Sporothrix schenckii*

Patricia Garcia Ferreira[a,b], Luana Pereira Borba-Santos[c], Leticia Lorena Noronha[a,b], Caroline Deckman Nicoletti[a,b], Marcella de Sá Haddad Queiroz[a,b], Fernando de Carvalho da Silva[a], Sônia Rozental[c], Débora Omena Futuro[b], Vitor Francisco Ferreira[a,b].

[a]*Universidade Federal Fluminense, Instituto de Química, Departamento de Química Orgânica, 24210-141, Niterói – RJ, Brazil.*

[b]*Universidade Federal Fluminense, Faculdade de Farmácia, Departamento de Tecnologia Farmacêutica, 24241-000, Niterói – RJ, Brazil.*

[c]*Universidade Federal do Rio de Janeiro, Instituto de Biofísica Carlos Filho, Laboratório de Biologia Celular de Fungos, 21941-902 - Rio de Janeiro, RJ – Brazil.*

Supplementary Material

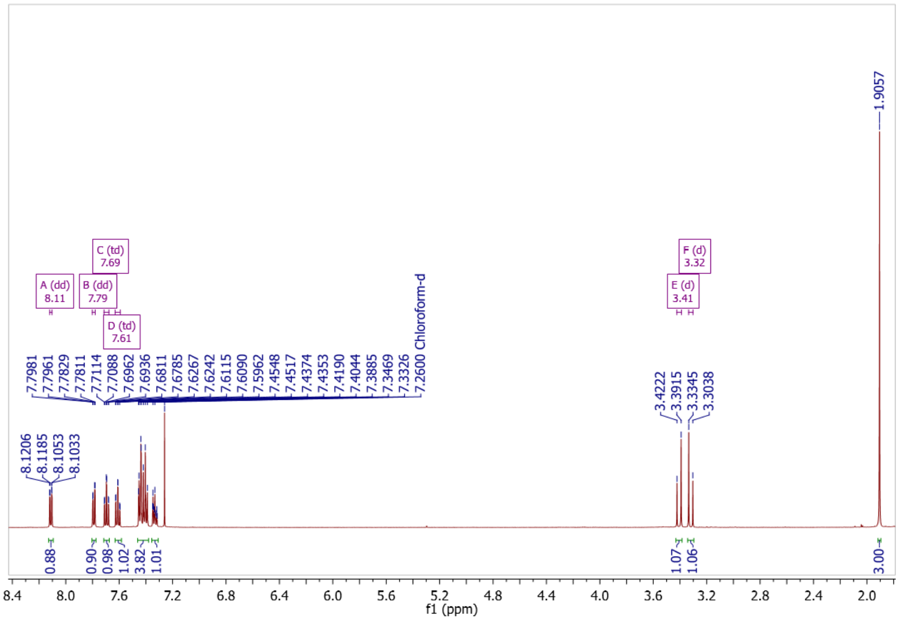

Fig.1: HNMR spectra of compound 10 after 60 days stored at -20°C.

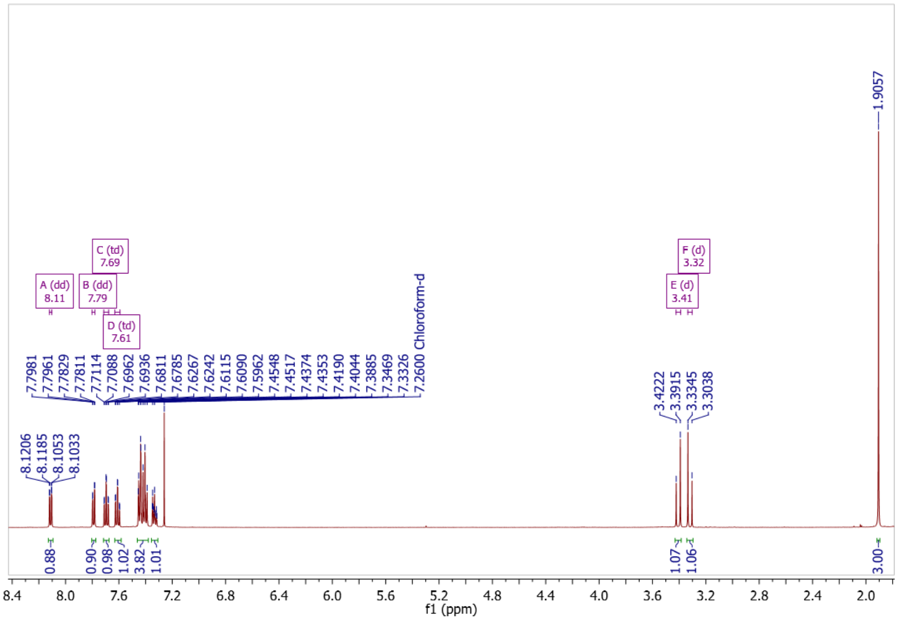

Fig.2: HNMR spectra of compound 10 after 60 days stored at 25°C.

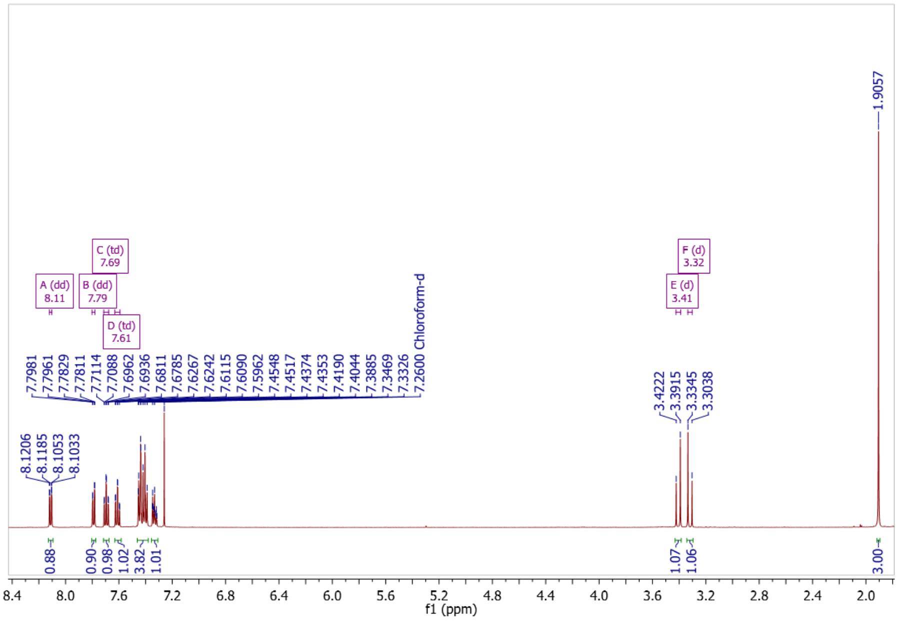

Fig.3: HNMR spectra of compound 10 after 90 days stored at -20°C.

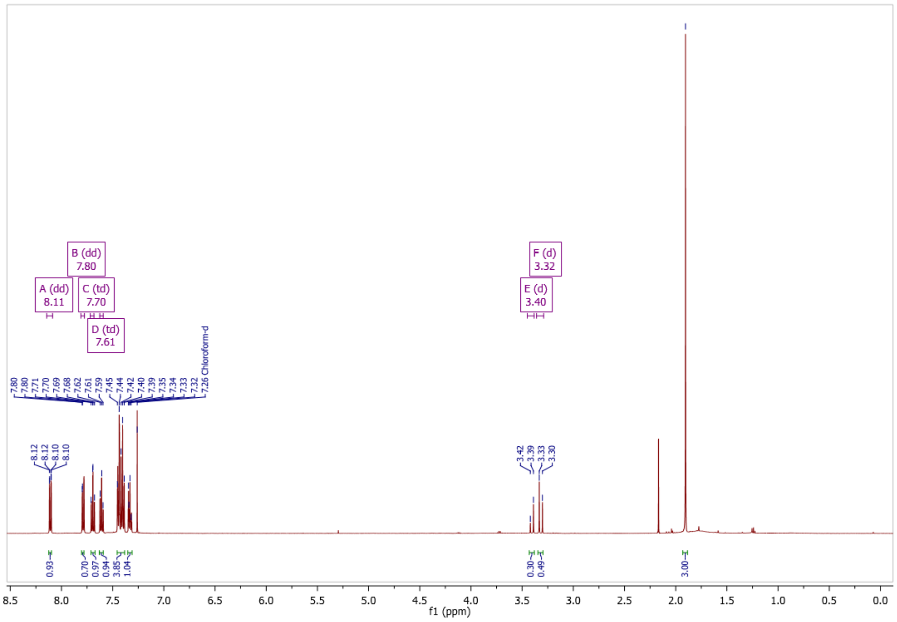

Fig.4: HNMR spectra of compound 10 after 90 days stored at 25°C.

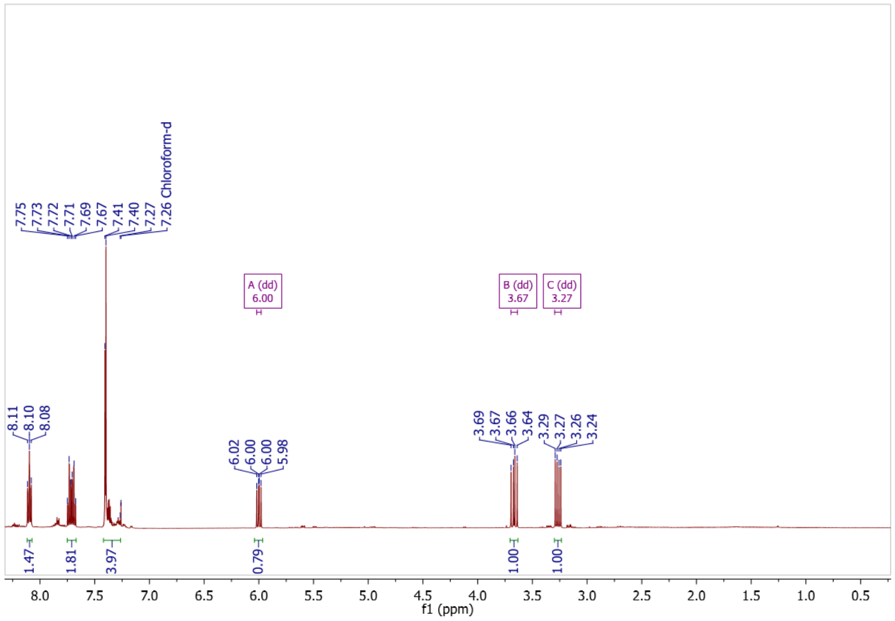

Fig.5: HNMR spectra of compound 6 after 60 days stored at -20°C.

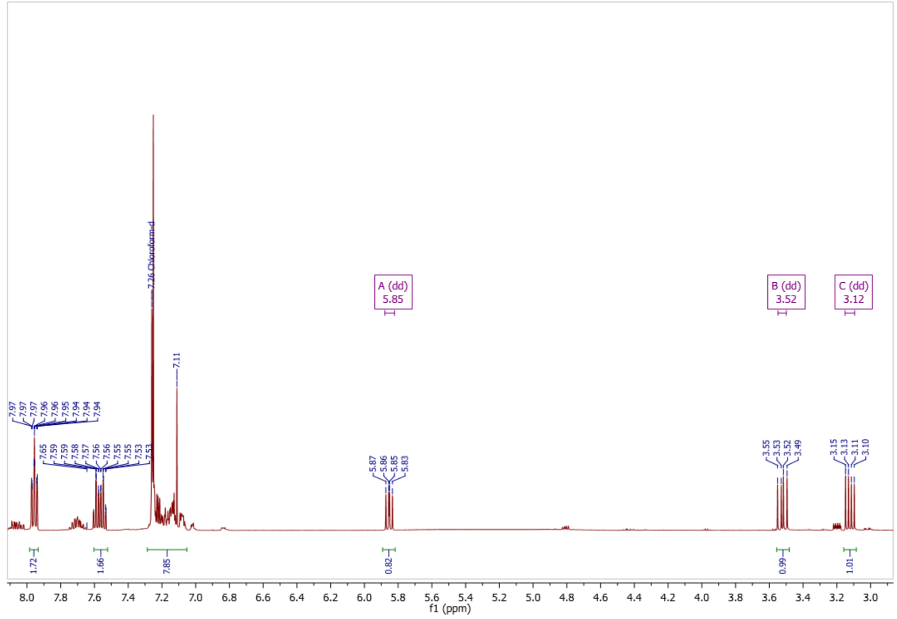

Fig.6: HNMR spectra of compound 6 after 60 days stored at 25°C.

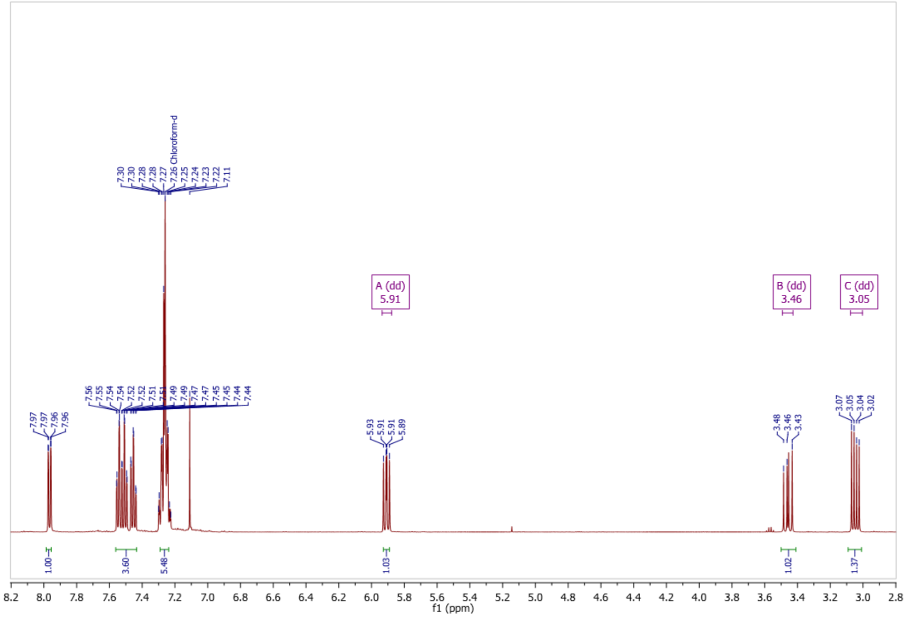

Fig.7: HNMR spectra of compound 6 after 90 days stored at -20°C.

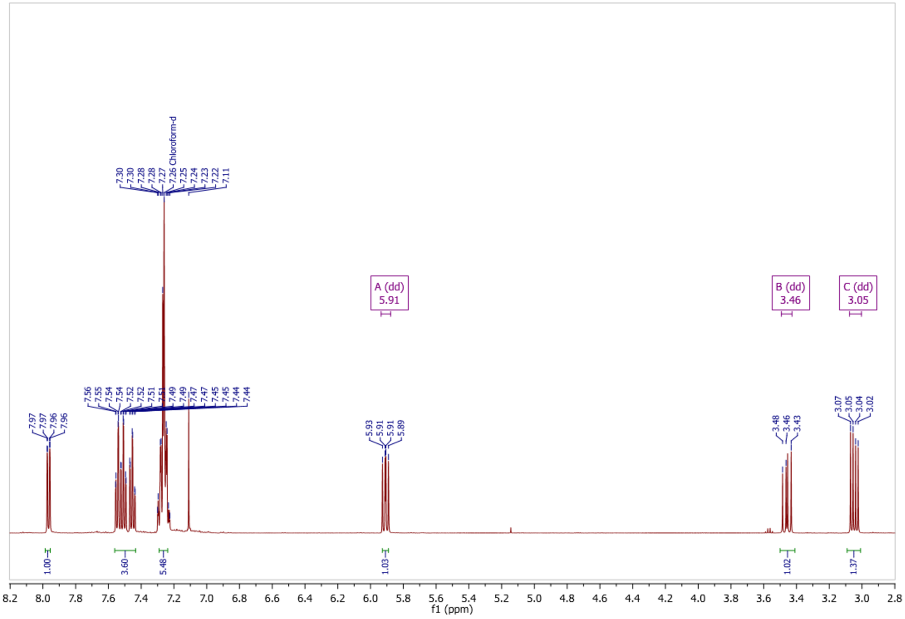

Fig.8: HNMR spectra of compound 6 after 90 days stored at 25°C.
